# Unveiling the Bioleaching Versatility of *Acidithiobacillus ferrooxidans*

**DOI:** 10.3390/microorganisms12122407

**Published:** 2024-11-23

**Authors:** Luca Tonietti, Mattia Esposito, Martina Cascone, Bernardo Barosa, Stefano Fiscale, Maria Teresa Muscari Tomajoli, Tomasa Sbaffi, Rosa Santomartino, Giovanni Covone, Angelina Cordone, Alessandra Rotundi, Donato Giovannelli

**Affiliations:** 1 Department of Science and Technology, University Parthenope, 80143 Naples, Italy; stefano.fiscale001@studenti.uniparthenope.it (S.F.); mariateresa.muscaritomajoli001@studenti.uniparthenope.it (M.T.M.T.); alessandra.rotundi@uniparthenope.it (A.R.); 2 International PhD Programme/UNESCO Chair “Environment, Resources and Sustainable Development”, 80143 Naples, Italy; 3 Department of Biology, University Federico II, 80126 Naples, Italy; mattiaesposito253@gmail.com (M.E.); martina.cascone1995@gmail.com (M.C.); bernard.barosa@gmail.com (B.B.); angelina.cordone77@gmail.com (A.C.); 4 INAF-OAC, Osservatorio Astronomico di Capodimonte, 80137 Naples, Italy; giovanni.covone@unina.it; 5 Molecular Ecology Group (MEG), National Research Council of Italy—Water Research Institute (CNR-IRSA), 28922 Verbania, Italy; tomasa.sbaffi@gmail.com; 6 UK Centre for Astrobiology, School of Physics and Astronomy, University of Edinburgh, Edinburgh EH8 9YL, UK; rosa.santomartino@ed.ac.uk; 7 Department of Physics, University of Naples Federico II, 80126 Naples, Italy; 8 INAF-IAPS, Istituto di Astrofisica e Planetologia Spaziali, 00133 Rome, Italy; 9 National Research Council, Institute of Marine Biological Resources and Biotechnologies, CNR-IRBIM, 60125 Ancona, Italy; 10 Department of Marine and Coastal Science, Rutgers University, New Brunswick, NJ 08901, USA; 11 Marine Chemistry & Geochemistry Department, Woods Hole Oceanographic Institution, Falmouth, MA 02543, USA; 12 Earth-Life Science Institute, ELSI, Tokyo Institute of Technology, Tokyo 152-8550, Japan

**Keywords:** *Acidithiobacillus ferrooxidans*, acidophiles, biomining, bioleaching, biorecovery, chemical elements

## Abstract

*Acidithiobacillus ferrooxidans* is a Gram-negative bacterium that thrives in extreme acidic conditions. It has emerged as a key player in biomining and bioleaching technologies thanks to its unique ability to mobilize a wide spectrum of elements, such as Li, P, V, Cr, Fe, Ni, Cu, Zn, Ga, As, Mo, W, Pb, U, and its role in ferrous iron oxidation and reduction. *A. ferrooxidans* catalyzes the extraction of elements by generating iron (III) ions in oxic conditions, which are able to react with metal sulfides. This review explores the bacterium’s versatility in metal and elemental mobilization, with a focus on the mechanisms involved, encompassing its role in the recovery of industrially relevant elements from ores. The application of biomining technologies leveraging the bacterium’s natural capabilities not only enhances element recovery efficiency, but also reduces reliance on conventional energy-intensive methods, aligning with the global trend towards more sustainable mining practices. However, its use in biometallurgical applications poses environmental issues through its effect on the pH levels in bioleaching systems, which produce acid mine drainage in rivers and lakes adjacent to mines. This dual effect underscores its potential to shape the future of responsible mining practices, including potentially in space, and highlights the importance of monitoring acidic releases in the environment.

## 1. Introduction

Extreme acidophilic microorganisms, which thrive in highly acidic conditions with a pH below 2.5 [1], are of particular importance in the biometallurgy context because they facilitate the mobilization of a diverse array of elements, including transition metals. This is achieved as a result of a spectrum of reactions involving various elements from the periodic table, through a process known as bioleaching [2,3,4,5]. Extremophiles are optimal microorganisms for developing mining applications on Earth [6,7,8], as well as in space [9,10,11]. For instance, as shown by Cockell et al. (2020), the extremophilic *Cupriavidus metallidurans* and the mesophilic *Sphingomonas desiccabilis*, through experiments with simulated Mars and Earth gravity on the International Space Station (ISS), have demonstrated key biomining capabilities of economically fundamental elements in space [9]. On the other hand, Tonietti et al. (2023) recently reported a shift in focus to acidophiles as the future avenue of space biomining, with *Acidithiobacillus ferrooxidans* being the prime candidate [11].

**Figure 1:**
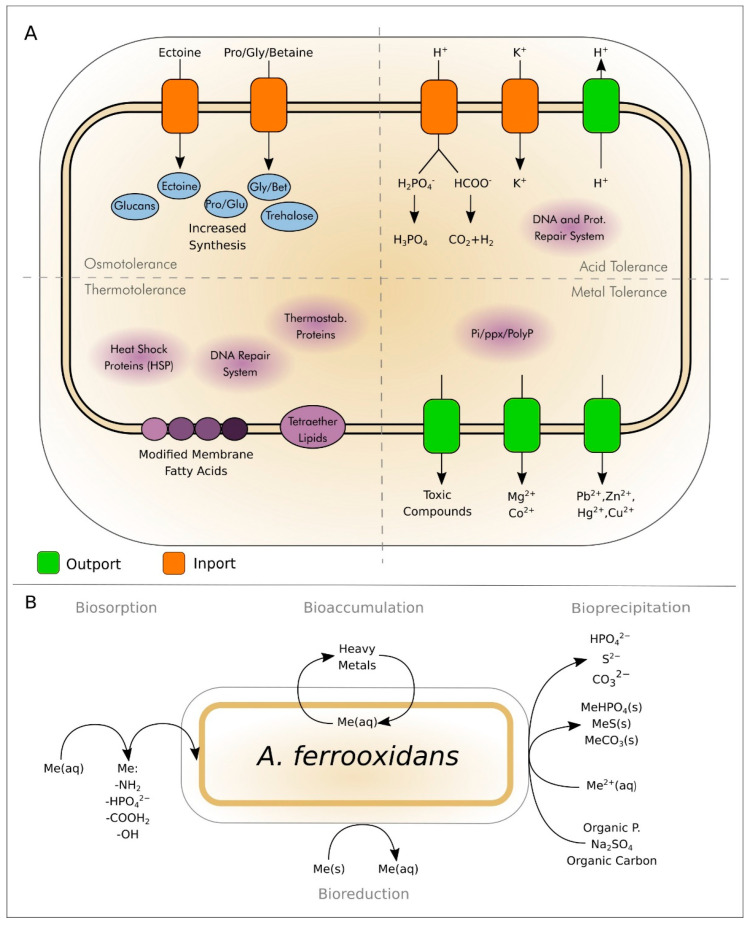
General mechanisms for acidophile tolerances and immobilization of metals from the environment. (**A**) In the upper left, osmotolerance with proteins and transporters. In the upper right, acid tolerance highlighting membrane transport proteins and the principal mechanisms involving proton transport. In the lower left, thermotolerance is displayed, and in the lower right, tolerance to heavy metals such as Pb, Zn, and Hg is illustrated. The main processes of metal immobilization found in acidophilic organisms such as *A. ferrooxidans*. (**B**) Clockwise from the top, bioaccumulation involves the internalization of heavy metals into the cell as aquo-ions, followed by bioprecipitation where hydrated metals are precipitated into their respective hydrogen phosphate, sulfide, and carbonate forms. Bioreduction entails the precipitation of soluble metals as solid metals through redox processes. Finally, biosorption processes involve the absorption of soluble metals by the cell in the form of solids, such as phosphates, or in compatible molecules, e.g., amines, organic acids, or hydroxides.

The Acidithiobacillaceae family, part of the Gammaproteobacteria class, encompasses acidophilic bacteria adept at surviving in extremely acidic environments [12]. These chemolithoautotrophs derive energy through the oxidation of inorganic compounds and fix carbon dioxide as their carbon source, significantly contributing to the biogeochemical cycling of sulfur and iron [13]. *A. ferrooxidans* and other members of the Acidithiobacillaceae family are widely acknowledged for their ability to mobilize a variety of elements, including metals and metalloids, amidst extreme conditions [12,14]. Their metabolism harnesses inorganic energy sources, such as Fe^2+^, H_2_S, S^0^, and molecular hydrogen, to sustain their metabolic activities [15] (Figure 1A).

Among the Acidithiobacillaceae, *A. ferrooxidans* (formerly known as *Thiobacillus ferrooxidans*) is notably well studied. This Gram-negative, rod-shaped gammaproteobacterium is renowned for its ability to oxidize ferrous iron to ferric iron and different reduced forms of sulfur compounds to sulfate. This organism was first isolated from the acidic water and sediments of a coal mine drainage in 1947. It was identified due to its ability to oxidize ferrous iron and sulfur compounds, leading to the production of acid which contributes to the acidic condition of the mine drainage environment. *A. ferrooxidans* thrives in highly acidic environments, typically within a pH range of 1.5 to 2.5. Its genome encodes enzymes critical for iron and sulfur oxidation, as well as mechanisms for adapting to acidic conditions [12]. The metabolism of the organism can be divided into different sections starting from the outer membrane, in which the process of iron oxidation occurs thanks to the presence of a specific electron transfer cytochrome c protein known as Cyc2. Many other different electron transfer molecules are used, such as the cytochrome c4, the cytochrome c552, the bc1 complex, and the cytochrome aa3 oxidase. All of these systems are part of the electron transport chain (ETC), in which electrons from Fe^2+^ are transferred to the inner membrane where the ETC is located. Within the oxidation of iron, *A. ferrooxidans* is also able to oxidize reduced sulfur compounds such as elemental sulfur, thiosulfate, and tetrathionate through different enzymes, e.g., the sox system (soxAX, soxB, soxCD, and soxYZ), sulfide oxidoreductase (SQR), and sulfur dioxygenase (SDO). Being an autotroph, the carbon source is the carbon dioxide (CO_2_), which can be used through the Calvin–Benson–Bassham cycle (CBB). Ecologically, Acidithiobacillaceae are predominantly found in environments such as acid mine drainage, sulfuric hot springs, and sulfide mineral deposits [16]. They are key players in biomining, facilitating the extraction of metals from ores through bioleaching. *A. ferrooxidans* is frequently associated with mine tailings and acidic drainage waters, contributing significantly to the oxidation of pyrite and other sulfide minerals [17], playing a crucial role in the formation and maintenance of acidic conditions. In these environments, *A. ferrooxidans* coexists with other hyperacidophiles, including other *Acidithiobacillus* species and members of the archaeal genera such as *Ferroglobus* and *Thermoplasma* [18]. Specifically, *A. ferrooxidans* has several adaptations to survive in these kinds of environments, such as acid-resistant proteins that are adapted to function optimally at low pH, the presence of proton pumps and antiporters to expel protons and maintain internal pH homeostasis, e.g., the presence of genes coding for K^+^/H^+^ transporting ATPase and the Na^+^/H^+^ antiporter gene, and the ability to produce biofilms on the mineral surface, providing a micro-environment that can help in pH regulation and nutrient acquisition [5].

The metabolic activities of these microorganisms are pivotal in bioleaching and biorecovery processes. *A. ferrooxidans*, along with its consortia partners, leads to the generation of sulfuric acid. This acid production is essential in the leaching of heavy metals from mine tailings, contributing to the environmental impact observed in mining areas [19,20]. The interactions between *A. ferrooxidans* and other hyperacidophiles in these consortia are complex and involve synergistic relationships that enhance the overall efficiency of mineral oxidation and acid production. Thanks to these kinds of processes, *A. ferrooxidans* is able to derive energy by oxidizing inorganic compounds that release electrons which can be transferred through the ETC, creating a proton motive force that drives the ATP synthesis.

## 2. General Bioleaching Process

**Figure 2:**
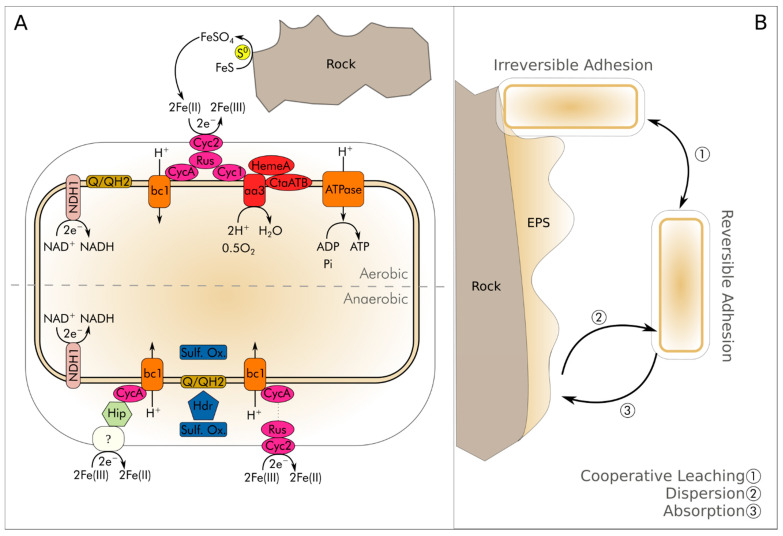
Mobilization mechanisms for *A. ferrooxidans*. (**A**) Aerobic and anaerobic protein pathway for iron oxidation and reduction. Under aerobic conditions, the transition from Fe(II) to Fe(III) occurs through hydrogen oxidation, NAD^+^ reduction, ATP production, and sulfur oxidation. In anaerobic conditions, the electron transport chain is mostly understood, except for an unidentified protein (?) responsible for the transformation of Fe(III) to Fe(II). (**B**) Planktonic and sessile cells permanently or non-permanently attached to the substrate. In the case of planktonic or floating cells, leaching occurs in the space between the rock and the organism via a series of redox reactions, while in the case of sessile cells, adherence to the substrate occurs due to extracellular polymeric substance (EPS) production, and leaching is termed direct or contact leaching. Rocks are generally sulfides, but not exclusively. Bioleaching can also be cooperative, wherein sessile and planktonic cells collaborate in metal mobilization between the acidic medium, substrate, and cells. Cell attachment to the substrate can be irreversible if EPS has already been produced, or reversible if not yet produced, and the process by which cells can adhere or detach from the substrate is termed absorption or dispersion.

*A. ferrooxidans* thrives in either aerobic or anaerobic conditions. Under aerobic conditions, Fe^2+^ and/or reduced sulfur compounds in the ores undergo oxidation, resulting in the formation of Fe^3+^ and sulfuric acid (H_2_SO_4_) [17]. In aerobic conditions (Figure 2A), the redox coupled reactions of *A. ferrooxidans*’s metabolism can be summarized from Equation (1) to Equation (5):

4Fe^2+^ + O_2_ + 4H^+^ → 4Fe^3+^ + 2H_2_O (1)

2H_2_ + O_2_ → 2H_2_O (2)

S^2−^ + 2H_2_O + 2O_2_ → SO_4_^2−^+ 4H^+^ (3)

2CO_2_ + O_2_ + 4H^+^ → 2HCOOH + 2H_2_O (4)

2NADH + O_2_ + 2H^+^ → 2NAD^+^ + 2H_2_O (5)

All of the above-mentioned reactions show the oxidation of different elements, e.g., Fe, H, S, CO_2_, and NADH coupled with the reduction of oxygen to produce water. The resultant oxidation products, Fe^3+^ and H_2_SO_4_ (Equations (1) and (3)), engage in chemical interactions with metal sulfide minerals and native metals within ores, leading to the dissolution and/or production of metal cations and the elimination of sulfur [21,22].

The conversion of sulfur/sulfide to sulfuric acid (Equation (3)) culminates in the formation of an intensely acidified medium, due to the production of H_2_SO_4_ [23]. This process facilitates the abiotic and biotic leaching of sulfides in mines, leading to the acidification of waters and the generation of acid mine drainage [24]. Moreover, oxidation and reduction processes can also be found in immobilization and mobilization processes (Figure 1B).

In the absence of oxygen (Figure 2A), *A. ferrooxidans* undergoes anaerobic growth, performing the reductive dissolution of ferric iron (Fe^3+^) oxy-hydroxide through the oxidation of elemental sulfur (S^0^ to S_2_O_3_^2−^). This reaction assumes pivotal significance in the “Ferredox” process, exemplified by the extraction of nickel from lateritic ores [25,26]. The major coupled reactions involved in the anaerobic metabolism can be summarized as follows [12,21]:

2Fe^3+^ + H_2_ → 2Fe^2+^ + 2H^+^ (6)

S^0^ + H_2_ → S^2−^ + 2H^+^ (7)

2Fe^3+^ + NADH → 2Fe^2+^ + NAD^+^ + H^+^ (8)

4Fe^3+^ 2S^0^ + 2H_2_O → 4Fe^2+^ + S_2_O_3_^2−^ + 4H^+^ (9)

2Fe^3+^ + CO_2_ + 2H^+^ → 2Fe^2+^ + HCOOH (10)

In natural environments, sulfide minerals undergo abiotic oxidation by the presence of O_2_ or chemical oxidants together with water or moist air [27]. Microorganisms instead utilize Fe^2+^ and sulfur for metabolic processes, leading to the formation of Fe^3+^ and sulfate ions. This metabolic activity results in a higher concentration of these ions, subsequently intensifying the dissolution of sulfide minerals and augmenting the volume of acidic water [28]. A quintessential illustration of this bioleaching phenomenon is the process involving chalcopyrite (CuFeS_2_) microbial-mediated dissolution (Equation (11)) [29]:

CuFeS_2_ + 2Fe_2_(SO_4_)_3_ → CuSO_4_ + 5FeSO_4_ + 2S^0^ (11)

which is driven by the following abiotic reactions (Equations (12)–(14)):

CuFeS_2_ + 2H_2_O + 3O_2_ → Cu^2+^ + Fe^2+^ + 2H_2_SO_4_ (12)

CuFeS_2_ + H_2_SO_4_ → Cu^2+^ + FeSO_4_ + 2H^+^ + 2S^0^ (13)

CuFeS_2_ + 4H^+^ + O_2_ → Cu^2+^ + Fe^2+^ + 2H_2_O + 2S^0^ (14)

In the presence of *A. ferrooxidans*, the FeSO_4_ produced by the abiotic reaction (Equation (13)) is oxidized at the expenses of oxygen in Fe_2_(SO_4_)_3_ by the microbe metabolism (Equation (15)), releasing further acidity that supports the overall reaction.

3FeSO_4_ + H_2_SO_4_ + 0.5O_2_ → Fe_2_(SO_4_)_3_ + FeSO_4_ + H_2_O (15)

The resultant primary sulfur product in the process of metal sulfide dissolution is related to the specific sulfide mineral being subjected to bioleaching, subsequently undergoing chemical or biological transformations into elemental sulfur and sulfate [30]. Disulfides, such as FeS_2_, MoS_2_, and WS_2_, can undergo oxidation through thiosulfate (S_2_O_3_^2−^) as the principal intermediate of the reaction [31]. Only Fe^3+^ ions, arising from the microbial oxidation of Fe^2+^, serve as oxidative agents for solubilization. The oxidation of S_2_O_3_^2−^ leads to the formation of SO_4_^2−^, with elemental S^0^ generated as a by-product. This elucidates why exclusively Fe^2+^-oxidizing bacteria possess the capability to oxidize these specific metal sulfides. Two main mechanisms contribute to the enhancement of metal leaching from mineral ores by microbial activity: direct and indirect action [31]. Direct action can be summarized as in Equations (16) and (17) (M is a bivalent metal):

MS + H_2_SO_4_ + 0.5O_2_ → MSO_4_ + S^0^ + H_2_O (16)

S^0^ + 1.5O_2_ + H_2_O → H_2_SO_4_ (17)

while indirect action uses Fe^3+^ as an oxidizing agent; microbes regenerate Fe^3+^ from Fe^2+^ (Equations (18) and (19)):

MS + 2Fe^3+^ → M^2+^ + 2Fe^2+^ + S^0^ (18)

2Fe^2+^ + 0.5O_2_ + 2H^+^ → 2Fe^3+^ + H_2_O (19)

The leaching of mineral sulfides in natural ecosystems is probably a combination of both direct and indirect mechanisms (Figure 2B) [32].

Bacteria, in the bioleaching of sulfide ores, can be momentarily or permanently connected to the mineral substrate, forming a biofilm which often plays a key role in the extraction process (Figure 2B) [33,34]. Extra-polymeric substances (EPSs) facilitate the adhesion of bacteria to metal sulfides, offering a survival advantage in adverse environments [35]. The adsorption affinity of *A. ferrooxidans* can be strengthened by hydrophilic substrates. This indicates that the geobiological affinity of the organism derives from the properties of the different substrates [36]. EPSs render apolar surfaces more polar, facilitating water infiltration through rocks and minerals. The EPSs primarily comprise neutral sugars and lipids, releasing H^+^ and Fe^3+^ ions [35]. The EPSs concentrate Fe^3+^ ions complexed with glucuronic acid at the mineral surface [37]. To summarize, in the direct mechanism (contact mechanism), *A. ferrooxidans* directly oxidizes the sulfide minerals or native metals, utilizing its enzymatic pathways to convert metal sulfides into soluble metal ions, e.g., sulfates. This process is facilitated by the bacteria’s ability to create EPSs. In the indirect mechanism (non-contact mechanism), *A. ferrooxidans* oxidizes ferrous iron to ferric iron from the environment and not directly from the mineral substrate. The ferric iron then acts as an oxidizing agent, attacking and solubilizing the sulfide minerals indirectly. This dual approach enhances the efficiency of bioleaching, making it a valuable method for metal extraction from environmental alkali cations and sulfate ions (Equation (20)):

3Fe^3+^ + 2SO_4_^2−^ + 6H_2_O + M^+^ → MFe_3_(SO_4_)_2_(OH)_6_ + 6H^+^ (20)

where M = K^+^, Na^+^, or NH_4_^+^. The precipitation of Jarosite (Equation (20)) is responsible for the passivation of certain minerals. The formation of a secondary mineral layer on the mineral surface serves as a diffusion barrier for reactant and product fluxes [38]. The principal contribution of *A. ferrooxidans* to metal extraction arises from its ability to convert insoluble sulfides into corresponding soluble sulfur–metal compounds [30].

*A. ferrooxidans* has potential applications in biorecovery, e.g., addressing challenges associated with abiotic acid mine drainage (AMD) production at mining sites and recovering heavy metals and other elements from contaminated sites [39]. Its efficacy in the removal of various metals from contaminated sources has been actively explored at the laboratory scale [40,41]. A mercury-resistant strain of *A. ferrooxidans* facilitated the recovery of mercury compounds from sediments and polluted fluids [42]. *A. ferrooxidans* has been employed in the bioremediation and biorecovery of Cr^6+^ and Cr^3+^ from electroplating waste [43]. The bacterium also plays a crucial role in arsenic bioremediation, leveraging its resistance mechanisms conferred by genes on its chromosome to participate in arsenic precipitation [44]. Regarding electronic waste (E-waste), the sulfuric acid produced by chemolithotrophic organisms, including *A. ferrooxidans*, serves as a leaching agent [45], increasing the acidity of the system and causing possible further releases of fundamental industrial chemical elements, e.g., lithium, into solution [46]. Due to this ability, the metals can be recovered with a feedback mechanism in which the acidity is increased, causing further acidification of the AMDs. This, on one hand, increases the risk of environmental damage, but on the other allows the recovery of toxic metals in confined systems.

### Chemical Elements Mobilized by A. ferrooxidans

Biomining and bioleaching are arising as some of the best tools in the context of In Situ Resource Utilization (ISRU) for space exploration, particularly utilizing extremophiles, e.g., *A. ferrooxidans*, or heterotrophic/chemolithoautotrophic organisms, e.g., *S. desiccabilis*, *B. subtilis*, or *C. metallidurans*. Bioleaching has been proposed as a technique for extracting valuable resources from regolith on Solar System bodies [10]. Regolith, a crucial in situ resource, contains elements such as oxygen, water, and trace metals that can help life-supporting devices and fuel production [47]. Bioleaching is a promising approach for extracting metals from regolith with a two-fold advantage for the space missions: (1) minimizing their cost, limiting the need for resources; (2) mitigating their logistical complexities [9,10]. Bioleaching in ISRU operations can contribute to optimizing the selection of landing sites in the frame of human planetary exploration and infrastructure development in space [48]. Biomining and bioleaching processes coupled with biosensors for space applications and new space technologies will be future methods to perform mineral extraction procedures on planets, asteroids, and moons [49,50,51]. All the techniques that have been proposed to be applied in space will need to rely on resources taken from the Earth, such as oxygen supply, nutrients, and chemicals to support microbial growth. Some of these can be obtained directly from the minerals present on other planetary bodies, while others must be brought from our planet.

Biomining and bioleaching present different ways to maximize economic performance in the mining industry. In the case of polymetallic ore, the achieved recoveries of metals are the following, Cu 90%, Au 90%, Ag 80%, Zn 72%, and Pb 90%, and the values per Net Present Value (NPV) and the Internal Rate of Return (IRR) are USD 21.927 million and 12.55%, respectively. Moreover, a report made by Credence Research (www.credenceresearch.com), an industry involved in data analysis in the changing global industry landscape, has shown that during the economic challenges of the pandemic (COVID-19), biomining showed revenues of USD 1500 million in 2020. Additionally, the projection for the future of biohydrometallurgical processes is promising, with a Compound Annual Growth Rate (CAGR) of over 13%. This trajectory positions biomining and bioleaching processes to reach an estimated value of USD 3600 million by 2027.

*A. ferrooxidans* is capable of mobilizing or affecting the mobilization of many different elements in the periodic table (Table 1), utilizing biochemical strategies depending on the specific element and the mineral/ore involved. The aim of this review is to report a comprehensive and updated list of elements which are mobilized by *A. ferrooxidans*, including its biochemical leaching mechanisms, and highlighting applications and limitations (Table 2).

## 3. Alkali Metals: Lithium

The alkali metals (Group 1 of the periodic table) constitute a highly reactive group of elements: lithium, sodium, potassium, rubidium, cesium, and francium. These metals share common characteristics: low melting points, high reactivity with water, tendency to form ionic compounds, and ability to readily donate electrons, leading to the formation of alkali metal cations with a + 1 charge [77]. Regarding bioleaching processes performed by the acidophile *A. ferrooxidans*, only lithium can be actively leached.

Being the lightest alkali metal, and due to its distinctive electrochemical reactivity, lithium finds extensive applications in metallurgy, aerospace, ceramics, battery technology, and fuel cells [78]. The utilization of Li-ion batteries for powering portable devices and electric vehicles has led to an escalating demand for lithium [79]. Naturally occurring sources of lithium include lake brines, pegmatites ore bodies, and sedimentary rocks, with over 80% of the current global production being derived from brines [80]. Developing technologies capable of extracting lithium from solid ores holds significant importance in addressing future lithium demand [81].

In comparison to brine extraction, the process of obtaining lithium from rocky sources is notably more intricate. This procedure encompasses a series of distinct operations, including roasting rocks in sulfate or carbonate environments to render lithium into water-soluble species [82].

One of the primary industrial minerals for lithium extraction is spodumene (LiAlSi_2_O_6_), a Li-bearing pyroxene found in pegmatites, i.e., coarse-grained igneous rocks rich in lithium, with a relatively low occurrence of other metals. Lepidolite (3Li_2_O*2K_2_O*5Al_2_O_3_*10SiO_2_*2SiF_4_), a form of pegmatite, member of the mica group, represents the most abundant lithium ore, with a distribution far surpassing that of Li-brines [83]. The Li_2_O content in lepidolite ranges from 3.0% to 7.7% by weight (1.39–3.58% Li), comparatively lower than that of spodumene, 6–8% by weight as shown by Sedlakova-Kadukova et al. (2020). Lepidolite exploitation in industrial processes is constrained by the elevated costs associated with lithium recovery from the mineral, necessitating high concentrations of acid in purification procedures [84]. The adoption of alternative technology has gained importance due to its potential for cost reduction, heightened efficiency, and environmentally sustainable processing [85]. A bio-extraction approach utilizing a consortium of different bacterial strains—namely, *A. ferrooxidans*, *A. thiooxidans*, as well as the heterotrophic fungus *Aspergillus niger* and yeast *Rhodotorula mucilaginosa*—has been investigated [86]. These microorganisms play integral roles in processes such as the bio-weathering of rocks, mobilization of metals from minerals, metal precipitation, and deposition [87]. For this reason, they have been extensively employed in biohydrometallurgical practices.

The mechanisms underlying lepidolite bioleaching by bacteria remain undisclosed. Nevertheless, it has been observed that, apart from H^+^ ions, no other substance contributes to the dissolution of Li^+^ ions. This implies that the dissolution of lithium in lepidolite is facilitated by acidic conditions [86,88]. Presumably, the primary reaction involved in the bioleaching of mixed alkali metals can be expressed through Equation (21), in which the metal oxide is converted into a soluble sulfate.

M_2_O + H_2_SO_4_ → M_2_SO_4_ + H_2_O (21)

where M presents alkali metals. Metals inside the mineral can be dissolved to form the sulfate counterpart, resulting in lepidolite dissolution [86]. The reaction of lepidolite bioleaching in sulfuric acid produced by bacteria can be seen in Equation (22):

3Li_2_O*2K_2_O*5Al_2_O_3_*10SiO_2_*2SiF_4_ + 20H_2_SO_4_ → 3Li_2_SO_4_ + 2K_2_SO_4_ + 5Al_2_(SO_4_)_3_ + 11SiO_2_ + H_2_SiF_6_ + 18H_2_O + 2HF (22)

In bacterial involvement, the composition of the medium emerged as the predominant factor influencing lithium bioleaching. The authors of [89] report that, within a nutrient-rich medium tailored for acidophilic chemoautotrophic acidithiobacilli, featuring essential energy sources in the form of ferrous ions and elemental sulfur, no lithium bioleaching was observed throughout the entire duration of the process. Conversely, in a medium with restricted nutrient content and limited energy sources, comprising solely sulfuric acid and elemental sulfur, the presence of Li^+^ ions was discernible within a matter of days. This suggests that bacteria, essential for lithium bioleaching from lepidolite, as no lithium was detected in abiotic control samples, may have been compelled to directly utilize nutrients vital for their sustenance from the leached material. Lithium extraction, achieved through bacterial intervention, necessitates at least 22 days to occur [86].

## 4. Non-Metals: Phosphorus

Non-metals encompass a variety of elements critical for chemical reactions and biological processes [90]. Non-metals, characterized by their lack of metallic luster, malleability and conductivity, such as oxygen, nitrogen, and carbon, are included in compounds essential for life, i.e., water, proteins, and DNA [91]. They contribute significantly to the Earth’s atmosphere, with oxygen being a crucial component for sustaining aerobic life [90]. Phosphorus (P) is the only non-metal of industrial interest bioleached by *A. ferrooxidans*, which can actually metabolize other non-metal elements such as carbon, nitrogen, and sulfur.

Phosphorus (P), a crucial plant nutrient, has attained increasing importance as a natural resource, given its finite supply [92]. The leaching of phosphorus into superficial waters poses a significant environmental challenge. Sewage sludge represents a substantial reservoir of phosphorus and, considering its substantial quantities generated globally daily, projections indicate a surge production attributable to population growth [93]. The disposal of such large volumes of sewage sludge raises substantial environmental apprehensions. Its application on agricultural land emerges as an attractive option, offering nutrient recycling and augmentation of soil organic content [94]. However, this approach confronts several constraints, as sludge (1) harbors numerous pathogens, (2) may contain elevated levels of heavy metals, and (3) is bulky and has associated transport costs, which pose logistical challenges [95].

Phosphorus recovery from sewage sludge in the form of phosphoric acid is facilitated by the use of sulfuric acid. Nevertheless, the process entails the costly steps of sludge drying and the utilization of significant quantities of commercial acid [96]. Active phosphorus extraction from P-sludge, deriving from the metabolic activities of acidophiles, is well connected with environmental recovery challenges, linking bioleaching and biorecovery. The bioleaching of rock phosphate employing acid-generating microorganisms has been extensively investigated [97,98]. The biochemical approach offers distinct advantages regarding eco-friendly approaches and biorecovery techniques [99]. The development of a biochemical process for phosphorus recovery from sludge is a highly advantageous endeavor.

*A. ferrooxidans* and *A. thiooxidans* present viable candidates for the recovery and bioleaching of P-rich sludge [100]. These microorganisms possess the capability to generate sulfuric acid through the oxidation of reduced sulfur compounds, allowing for the establishment of an acidic pH level, potentially below 1. This produced sulfuric acid serves to solubilize the phosphorus content. *A. thiooxidans* demonstrates the capacity to recover 94% of phosphorus from rock phosphate, while *A. ferrooxidans* can be employed to solubilize phosphorus from desiccated sewage sludge [101]. The chemical reactions governing the leaching of phosphorus from the sludge are delineated by Equations (23) and (24):

3H_2_SO_4_ + Ca_3_(PO_4_)_2_ → 2H_3_PO_4_ + 3CaSO_4_ (23)

3H_2_SO_4_ + 2FePO_4_ → 2H_3_PO_4_ + Fe_2_(SO_4_)_3_ (24)

The process takes circa 63 days to recover 92% of the phosphorus from the sludge. The extracted phosphoric acid can be used for different purposes, including the production of ammonium phosphate [101]. Being a technique based on microorganisms and not on expensive industrial processes, the operation is time-consuming but allows the recovery of phosphorus from wastes.

## 5. Transition Metals

Transition metals exhibit distinctive electronic configurations, with partially filled d-orbitals contributing to their characteristic properties. Their ability to form stable coordination complexes makes them essential in fields such as catalysis, where they play a crucial role in accelerating chemical reactions [102]. Transition metals are known to be fundamental to many organisms; in microbes, they drive the global biogeochemical cycling of elements [103,104]. Today, 25% of the total transition metals can be mobilized by *A. ferrooxidans*.

### 5.1. Vanadium

Vanadium (V) represents a prototypical redox-sensitive transition metal widely employed in industries, including metallurgy, manufacturing, and petroleum refining [105]. Vanadium serves as a catalytic agent in denitrification processes, acting as a metallic cofactor within enzymes responsible for catalyzing such reactions [106]. Vanadium redox batteries present a further potential application in addressing the escalating global energy demand [107].

A significant proportion of the world’s vanadium production stems from slags generated during steelmaking operations employing V-titanium magnetite [108]. Conventional methods for vanadium extraction are marked by high costs and inefficiencies, accompanied by pronounced environmental challenges, encompassing alkali melting, chloridizing roasting, acid leaching, water leaching, and deposition [109]. Bioleaching has been successfully employed for the solubilization of vanadium from solid waste sources, including spent refinery catalysts and oil-fired ash [110], and thus, its integration with established processes holds considerable promise. Successful bioleaching of vanadium through heterotrophic organisms has been demonstrated in microgravity conditions aboard the International Space Station (ISS) [111]. The incorporation of vanadium into steel enhances critical attributes such as hardness, tensile strength, and fatigue resistance, thereby elevating overall performance. Therefore, vanadium holds a key role in future space exploration by its possible use in the manufacturing of rovers, pressurized buildings, superconducting materials, batteries, and alloys for nuclear reactors. Being sufficient on Earth for the next few decades or centuries, it would be fundamental to find a way to extract it locally on other planetary bodies to reduce the environmental and economic cost associated with space transportation [111].

Minerals conducive to vanadium extraction encompass V-titanium magnetite ((V, Ti),Fe^2+^Fe^3+^_2_O_4_), maghemite (a specific variant of magnetite characterized by a low Fe^2+^ concentration), bixbyte (containing vanadium along with (Mn, Fe)_2_O_3_)), vanadium oxide, and jacobsite, typically (Mn, Mg)Fe_2_O_4_ [52]. A prominent participant in the interaction with these mineral types for vanadium extraction is *A. ferrooxidans*, catalyzing the conversion of Fe^2+^ to Fe^3+^ under acidic conditions (Equation (19)); following the oxidation of iron, the resulting Fe^3+^ can be used to oxidize vanadium in Equations (25)–(27) [112,113]:

V_2_O_3_ + 2Fe^3+^ + 2H^+^ →2VO^2+^ + 2Fe^2+^ + H_2_O (25)

VO_2_ + Fe^3+^ → VO_2_^+^ + Fe^2+^ (26)

10VO_2_ + 10Fe^3+^ + 8H_2_O → HV_10_O_25_^5−^ + 10Fe^2+^ + 15H^+^ (27)

Presumably, V^5+^ represents the predominant speciation in the leachate. Vanadium manifests three principal valence states in natural aqueous solutions, with V^5+^ prevailing as the most prevalent form in oxidizing environments [114]. V^5+^ can be readily retrieved from the leachate through NH_4_Cl precipitation [115]. In summary, *A. ferrooxidans* proves highly effective in the recovery of vanadium from both raw minerals and intermediate materials utilized in vanadium extraction processes. The average recovery efficiency is estimated to be approximately 30% [113].

### 5.2. Chromium

Chromium (Cr) slags represent a category of hazardous waste characterized by the prevailing toxicity of Cr^6+^. This hexavalent chromium species possesses a robust oxidizing potential toward living cells and has been associated with gene mutations and carcinogenic effects in animal studies (Sharma et al., 2022 [57]). In specific chromium slag compositions, such as Ca_3_Al_2_(H_4_O_4_, CrO_4_)_3_ and Ca_4_Al_2_(OH)_12_(CrO_4_)_3_*26H_2_O, approximately 20–25% of the total mass is constituted by hexavalent chromium [58]. Chromium salts are extensively employed in the leather industry for tanning processes, a treatment that safeguards leather against various environmental stressors, including microbial degradation and moisture [116]. An estimated 90% of tanneries employ chromium salts as a principal tanning agent. Chromium exhibits limited biodegradability and tends to accumulate within biological organisms, resulting in severe health ailments. Disposal methods for tannery sludge encompass incineration, solidification, landfills, and ocean dumping, with the last two facing mounting scrutiny in numerous countries due to environmental considerations [59].

The recovery of chromium from sludge can be facilitated through the action of sulfur- and iron-oxidizing bacteria. This microbiological approach enables the ready solubilization of metals via the oxidation of metal sulfides into soluble sulfates, or through acid dissolution employing sulfuric acid [117]. Subsequently, released chromium can be efficiently removed. Chromium is naturally occurring in minerals such as the Cr-bearing phase of goethite Fe^3+^O(OH), or in limonite deposits [118]. The liberation of chromium from sludge or rocks is contingent upon redox processes. The indirect activity of *A. ferrooxidans* mediates the oxidation of iron from Fe^2+^ to Fe^3+^, thereby facilitating the reduction of Cr^6+^ to the less toxic and water-insoluble Cr^3+^ species, increasing biorecovery processes [119]. In this process, Fe^2+^, reduced sulfur, and organic matter constitute the principal sources of electrons. These electrons released by the oxidation of iron through indirect mechanisms carried out by *A. ferrooxidans* are able to reduce the chromium in solution, leaving it free to be recovered in industrial processes. The reduced Fe^2+^ can serve as a reducing agent for Cr^6+^. The Fe^3+^ formed in the oxidation of Fe^2+^ is capable of accepting electrons from Cr^6+^, thereby reducing chromium. The Fe^3+^ can be further reduced back to Fe^2+^ by the microbial activity or other reducing agents present in the environment, maintaining a cycle that can continuously facilitate the reduction of Cr^6+^.

Cr^6+^ + 3Fe^2+^ → Cr^3+^ + 3Fe^3+^ (28)

The reduction of chromium under experimental conditions [120] is a process that iron-oxidizing bacteria, such as *A. ferrooxidans*, *A. thiooxidans*, and *P. putida*, can mediate.

### 5.3. Iron

Iron (Fe) is a fundamental element in various industrial processes, serving as a critical component in the production of steel, alloys, and numerous other materials essential for modern infrastructure. The demand for iron in industry underscores the significance of efficient extraction methods. Iron is also fundamental in household applications, e.g., refrigerators, stoves, washing machines, and packaging [121].

*A. ferrooxidans* exhibits the ability to oxidize ferrous iron to ferric iron, a process crucial for the solubilization of iron minerals in ore deposits. This metabolic proficiency includes iron oxidases, cytochromes, and electron transport chains within the bacterial cell. The unique redox chemistry of *A. ferrooxidans* enables it to thrive in highly acidic environments, where conventional mining operations are impractical [22]. The oxidation of ferrous iron by *A. ferrooxidans* can be seen in Equation (19).

The use of *A. ferrooxidans* in iron biomining from pyrite and iron sulfides, such as bornite (Cu_5_FeS_5_), offers several significant environmental advantages. Unlike traditional mining practices, which often result in extensive environmental degradation and habitat destruction, biomining is a less-impacting process [122]. By harnessing the metabolic capabilities of *A. ferrooxidans*, the extraction of iron from ore deposits can be achieved with minimal disturbance to surrounding ecosystems in a controlled environment. The reduction in chemical reagents and energy consumption associated with biomining contributes to lower carbon emissions compared to conventional mining techniques [39].

The application of *A. ferrooxidans* in iron biomining holds immense potential for the mining industry. With advancements in biotechnology and microbial engineering, there is a growing opportunity to optimize the efficiency and scalability of this process [123]. Ongoing research efforts are focused on expanding the repertoire of biomining microorganisms, potentially unlocking new niches for the extraction of valuable metals from diverse mineral resources [14].

The unique metabolic capabilities of *A. ferrooxidans* in iron biomining hold promise for extraterrestrial applications. In space exploration, the availability of essential resources such as iron is of paramount importance for construction, habitat development, and various technological endeavors. Leveraging *A. ferrooxidans* in biomining processes could facilitate the sustainable extraction of iron from planetary bodies, reducing reliance on resource-intensive transportation from Earth [11].

### 5.4. Nickel

Nickel (Ni) plays a prominent role in the manufacturing of electronic devices, which are subject to heightened demand driven by planned obsolescence and rapid market penetration. This results in a substantial accumulation of electronic waste (E-waste), which constitutes a vital reservoir of metals relevant for industry and rare elements [124]. Printed circuit boards (PCBs) account for approximately 3% of the total E-waste mass [125]. PCBs encompass notable quantities of Cu (10–20%), Ni (1–3%), Fe (1–4%), and Pb (1–5%), as well as precious metals (0.3–0.4%) including Au, Ag, and Pt, rendering them a valuable secondary metal resource [126]. Metal recovery from E-waste stands as a more sustainable and resource-efficient endeavor in terms of energy utilization, environmental protection, and overall sustainable resource management [125]. While hydrometallurgical and pyrometallurgical processes present notable drawbacks, e.g., concentrated acidic effluents, high energy consumption and big volumes of hazardous waste, [127,128], bioleaching contributes to the conservation of non-renewable energy [129].

Nickel and its compounds exert detrimental effects on the human heart and liver [130]. Coupling nickel’s hazards with its high demand in the electronics industry, bioleaching emerges as a promising method for its recovery from waste sources. In oxygen-rich environments and under acidic conditions, *A. ferrooxidans* through the oxidation of iron (Equation (19)) is able to produce Fe^3+^, which can be used in nickel oxidation [60].

In this manner, Ni^0^ present in E-waste undergoes oxidation through ferric ions, which are subsequently reduced to ferrous ions as in Equation (29):

Fe_2_(SO_4_)_3_ + M^0^ → M^2+^ + SO_4_^2−^ + 2FeSO_4_   M[Cu, Ni, Ga] (29)

In this specific case, M is nickel. The ferrous ions are oxidized back to ferric ions through *A. ferrooxidans* activity: a recurrent iron cycle is instituted within the nickel bioleaching process, whose efficiency depends on pH adjustment (88.9% with pH adjustment vs. 92.1% without it) [131]. Arshadi and Yaghmaei (2020) demonstrated the generation of jarosite and goethite, [60] see Equation (20).

### 5.5. Copper

Copper (Cu), a vital element in various industries, including electronics, construction, and transportation, necessitates efficient extraction methods to meet global demands. It is a fundamental material for electrical wiring, power transmission, and electronic components due to its superior conductivity, making it essential for powering homes, industries, and electronic devices [61]. Copper is also widely employed in plumbing, roofing, and architectural elements. Its corrosion resistance and durability make it a preferred choice for water pipes, roofing materials, and decorative features in buildings. Copper is used in transportation and the automotive industry and intensely in industrial machinery and equipment [132]. The presence of Fe^2+^ acts as an electron donor in this redox process, enabling the sustenance of *A. ferrooxidans* in highly acidic environments [133]. The bioleaching of copper minerals, e.g., chalcopyrite, can be represented by Equations (11)–(14) (see also Table 1). By harnessing the metabolic capabilities of *A. ferrooxidans*, the extraction of copper can be achieved with reduced ecological impact. The decreased dependence on harsh chemical reagents and energy-consuming methods results in a smaller environmental footprint relative to traditional mining techniques [134].

Biohydrometallurgy processes, including bioleaching, account for approximately 20–25% of the world’s copper production [135]. These processes involve the microbial oxidation of sulfide minerals, converting insoluble metal sulfides into soluble metal sulfates according to Equation (11). This approach is particularly efficient for low-grade ores, which are economically unfeasible to process using traditional methods. Traditional methods, such as smelting, require high energy input and produce significant environmental pollution. In contrast, bioleaching operates at ambient temperatures and pressures, utilizing natural microbial processes to extract metals with minimal environmental disruption [136].

The efficiency of bioleaching in low-grade ore deposits makes it an invaluable tool for mining operations. These deposits, often overlooked due to their low metal content, become viable sources of copper through bioleaching. This not only maximizes the utilization of available resources but also minimizes waste and environmental impact, aligning with sustainable mining practices [137]. Consequently, bioleaching with *A. ferrooxidans* represents a cost-effective, environmentally friendly alternative to conventional methods, contributing significantly to the global supply of copper.

Copper can also be extracted from many different minerals such as chalcocite (Cu_2_S) and covellite (CuS), with a total copper recoveries of 65–80%, and from enargite (Cu_3_AsS_4_), with a recovery rate between 7.3 and 27.1%. Furthermore, the addition of particular cations to the reaction e.g., Ag^+^, Hg^2+^, Bi^2+^, Cu^2+^, and Co^2+^, can decrease the time required for the bioleaching process. They reported that the majority of studies are focused on Ag^+^ in copper-bearing ore and chalcopyrite leaching. The addition of Ag^+^, at a catalytic concentration, led to the formation of a thin layer of silver sulfide (Ag_2_S) on the mineral surface, minimizing passivation throughout the process and enhancing electron transfer to the oxidant. The chemical reactions behind this process can be seen from Equations (30)–(32):

CuFeS_2_ + 4Ag^+^ → Cu^2+^ + Fe^2+^ + 2Ag_2_S (30)

2Ag_2_S + 4Fe^3+^ → 4Ag^+^ + 4Fe^2+^ + 2S^0^ (31)

CuFeS_2_ + 4Fe^3+^ → Cu^2+^ + 5Fe^2+^ + 2S^0^ (32)

Elemental silver can also be formed as in Equation (33):

CuFeS_2_ + 4Ag^+^ → Cu^2+^ + Fe^2+^ + 4Ag^0^ + 2S^0^ (33)

The main role of *A. ferrooxidans* in this process is the conversion of Fe^2+^ to Fe^3+^, which is capable of oxidizing the Ag_2_S layer.

In general, the addition of 7.1 g of Ag^+^ per Kg of minerals to the reaction led to a final yield of recovery of 89.3% of Cu compared to the traditional one of 28% without a catalyst. Similar results can be also obtained for Hg^2+^ (80% recovery after addition of the cation compared to 25% without), and Bi^2+^ (90% vs. 80% recovery ratio) [61,132,133,134,135,136,137].

### 5.6. Zinc

Zinc (Zn) is a critical element in the galvanizing, cosmetic, die-casting, and manufacturing sectors. With a global production exceeding 8 × 10^6^ tons, zinc is primarily extracted utilizing sulfide ores through hydro-metallurgical processes (roasting, leaching, and electro-winning), with the leaching kinetics playing a key role in the smelting procedure [138]. Zinc sulfide ores, especially sphalerite (ZnFe)S, are suitable and useful sources for Zn extraction as they are easily separated and concentrated by flotation from waste rocks [139]. Due to the increasing demand for zinc and Zn compounds, new zinc sources, e.g., low-grade oxide ores, have been considered.

The leaching of zinc ores with sulfuric acid has been investigated [140]. The zinc general chemical mobilization from ores can be summarized following Equation (16), in which M can be substituted by zinc.

Fowler and Crundwell (1998) experimentally disentangled two *A. ferrooxidans* zinc extraction mechanisms, direct and indirect, demonstrating that the indirect mechanism plays a central role in the leaching of zinc sulfide [141].

### 5.7. Molybdenum

Molybdenum (Mo) is an important element in sectors such as metallurgy, aerospace, and electronics [142]. Molybdenum is known for its exceptional heat resistance and high melting point, making it a crucial component in alloys that withstand extreme temperatures. Its ability to facilitate electron transfer makes it indispensable in electronics and electrical applications. Molybdenum finds extensive use in the production of steel and other alloys, contributing to enhanced strength and corrosion resistance [143]. Given its increasing demand, efforts have been made for the recovery of molybdenum from waste sources, contributing to sustainable resource management [144]. Bioleaching presents a promising avenue for molybdenum recovery: *A. ferrooxidans* has shown potential in solubilizing molybdenum from various sources, offering advantages in terms of safety, environmental sustainability, and energy efficiency [145].

Molybdenum is oxidized from Mo^4+^ to Mo^6+^ through the activity of *A. ferrooxidans* (Equation (34)). The ferrous ions released from Equation (34) are further oxidized to ferric ions, establishing a recurring iron cycle within the process. The soluble molybdenum can then be readily extracted, contributing to the efficient recovery of molybdenum from waste materials [145].

4MoS_2_ + 4Fe^3+^ + 16H_2_O → 4MoO_4_^2−^ + 4Fe^2+^ + 32H^+^ + 8S^0^ (34)

This reaction leads to the dissolution of molybdenum sulfide minerals, releasing molybdenum ions (MoO_4_^2−^) into the solution [146]. *A. ferrooxidans* contributes to the precipitation and immobilization of molybdenum through biomineralization processes [145]. Under appropriate conditions, molybdenum ions in solution can react with other elements to form various Mo-containing minerals. For example, the reaction between molybdenum ions and calcium ions (Ca^2+^) can lead to the formation of calcium molybdate CaMoO_4_ [147].

### 5.8. Tungsten

Among others metal ions (iron, copper, nickel, zinc, and molybdenum) tungsten (W) serves as an essential component of various metalloenzymes, playing pivotal roles in biological processes [104]. Some bacteria have evolved the capability to synthesize metal-binding proteins, facilitating the storage of these crucial metal ions within the cell [148]. Tungsten exhibits multiple oxidation states (e.g., W^6+^, W^4+^, W^2+^, and W^0^), with limited knowledge regarding its speciation in natural environments. Wolframite—(Fe,Mn)WO_4_—an iron, manganese, and tungstate mineral, alongside scheelite (CaWO_4_), represents a primary source of tungsten ore [65]. Wolframite undergoes instability in acidic and oxidizable conditions, leading to the formation of H_2_WO_4_ as the prevalent secondary tungsten mineral [66]. In acidic and reducing environments, the dominant tungsten species are W^4+^ and metallic W^0^. The leaching process of wolframite is a two-step reaction (Equations (35) and (36)), occurring after the oxidation of sulfides and necessitating interaction with the product of the first step of the reaction, H_2_SO_4_ [67].

H_2_SO_4_ + FeWO_4_ → FeSO_4_ + H_2_WO_4_ (35)

H_2_SO_4_ + MnWO_4_ → MnSO_4_ + H_2_WO_4_ (36)

The activity of acidophiles can result in the release of natural organic compounds, which have the potential to significantly enhance the mobility of tungsten in the environment [149]. These organic compounds can co-precipitate with ferrihydrite, Fe(OH)_3_, across a wide pH range (from 4 to 11), potentially interfering with the interaction between soluble tungstate oxyanions (WO_4_^2−^) and ferrihydrite [150]. The activity of acidophiles plays a pivotal role in the decomposition of wolframite and the mobilization of tungsten in mining waste environments [151]. During the initial stages of wolframite weathering under acidic conditions, free tungstate ions may bind with various cations, giving rise to a diverse array of secondary tungsten minerals, e.g., gallium-rich tungstate and sanmartinite (Zn,Fe)WO_4_ [152]. This phenomenon could potentially restrict the availability of tungsten in the environment.

The activity of *A. ferrooxidans* and acidophilic organisms holds significant potential for bioleaching tungsten minerals, primarily wolframite. The efficiency of this process is highly contingent on the microbial composition of the environment and the specific speciation of tungsten, which is thermodynamically influenced by the in situ redox state and other environmental variables [66].

### 5.9. Gold

Gold (Au) has played a key role in the development of human civilization, as the basis for decorative, ceremonial, and religious artifacts. Due to the physical and chemical properties of gold, such as its ability to not chemisorb oxygen, or the fact that it does not corrode, makes it an excellent catalyst. It is widely used in industrial applications by both homogeneous and heterogeneous gold catalysis [153]. The recovery of gold from rock mostly relies on the cyanidation process, Equation (37):

4Au^0^ + 8NaCN + 2H_2_O + O_2_ → 4NaAu(CN)_2_ + 4NaOH (37)

The use of cyanide for the dissolution of gold poses harsh environmental risks while bioleaching for gold recovery reveals promising avenues for more sustainable practices. Cyanide-producing bacteria autonomously convert cyanide to the less toxic form B-cyanoalanine [154]. *A. ferrooxidans* in the bioleaching of gold is part of the indirect method towards the elimination of interfering metal sulfides from ores containing precious metals. In gold ores, minute gold particles are encapsulated or tightly bound within a sulfide mineral, typically pyrite, arsenopyrite, or a combination of both [68]. To achieve satisfactory gold recovery, it is important to first oxidize the sulfide minerals prior to subjecting the ore to cyanide treatment for gold dissolution [69,155].

### 5.10. Mercury

Mercury (Hg) is produced globally, with the European contribution led by the Spanish mining industry. The Americas and Asia, particularly Kyrgyzstan, Russia, and China, also play significant roles in mercury extraction [156]. The 20th century saw a spike in global production, driven by industrial use, especially in the chlor-alkali industry, where mercury cells are used for electrolysis [157,158]. Beyond industry, mercury has been used in dentistry for over a century, with dental amalgam making up a significant portion of its usage in the USA and Sweden. Despite alternatives and known environmental impacts, international efforts against Hg in dental amalgam are lacking [158].

Cinnabar (HgS) has a long history of use as a natural pigment and in medicine and preservation for thousands of years [159]. The dissolution of cinnabar was found to be closely related to iron concentration, particularly influenced by ferric iron generated by *A. ferrooxidans* [160]. Although natural oxidation of cinnabar is limited [161], ferric iron catalyzes the reaction as in Equation (38):

HgS + 2Fe^3+^ → Hg^2+^ + 2Fe^2+^ + S^0^ (38)

During prolonged reactor operation with immobilized cells, it has been observed that a significant number of cells emerge into the liquid medium; this process has been explained by the adhesion of *A. ferrooxidans* on sulfur particles, with it becoming hydrophilic [162]. Thermodynamically, the free energy of adhesion is determined by interfacial tensions between bacteria, liquid, and sulfur phases [163]. Hydrophobic secretion by *A. ferrooxidans* enhances contact and adhesion [164]. The adhered sulfur particles are further oxidized by *A. ferrooxidans* (Equation (17)). This reaction inhibited ferric iron hydrolyzation [165], maintaining oxidant (ferric iron) concentration [166]. Substituting this into the cinnabar oxidation equation in Equation (39) yields the following:

2HgS + 4Fe^3+^ +1.5O_2_ +2H_2_O → 2Hg^2+^ + 4Fe^2+^ + 2H_2_SO_4_ (39)

*A. ferrooxidans* rapidly oxidizes ferrous iron. The oxidation process led to a reduction in H^+^ concentration, thereby aiding in the dissolution of cinnabar. The detected change in surface area pre- and post-bioleaching suggested a smoother particle surface prior to the reaction, which subsequently became more fragmented. This transformation was attributed to the indirect catalytic effect of ferric iron produced by *A. ferrooxidans* and the direct oxidation function that supplied energy for the growth of the bacteria [162]. By utilizing *A. ferrooxidans*, the toxic cinnabar can be broken down, reducing its environmental impact and recovering the mercury present in it.

## 6. Post-Transition Metals

Beyond the classical distinction between metals and nonmetals, a diverse group known as post-transition metals comprise elements found between the transition and metalloid regions. Post-transition metals, including aluminum and gallium, showcase a diversity of properties, encompassing malleability, conductivity, and varied oxidation states [167]. Their presence in alloys, combined with their unique reactivity patterns, makes them essential in the manufacturing of diverse materials, from lightweight alloys to corrosion-resistant coatings [168].

### 6.1. Gallium

The demand for Gallium (Ga) is projected to undergo rapid escalation from 2014 to 2050 [62,169] due to development of light-emitting diodes (LEDs) [63]. Bioleaching presents distinct advantages for Gallium recovery, characterized by its enhanced safety, environmental friendliness, and energy-efficient processes, enabling the leaching of metals even at relatively low concentrations. An adapted strain of *A. ferrooxidans* demonstrated the capacity to leach approximately 60% of the gallium content from powdered LEDs [170]. The efficacy of *A. ferrooxidans* for augmenting gallium bioleaching from LED powder establishes a suitable path for gallium recovery from LED waste [171]. The general bioleaching process can be summarized following Equation (29), in which M is gallium.

### 6.2. Lead

Lead (Pb) holds a long history of human use dating back many centuries. Its malleability and resistance to corrosion have made it a valuable metal for various applications, including pipes and paints, since the time of the Romans [172]. Lead found application e.g., in lead glazes for pottery and insecticides, despite being recognized for its carcinogenic properties; it is still utilized in the production of car batteries, pigments, ammunition, cable sheathing, radiation shielding, and soldering [72]. The primary mineral source of lead is galena (PbS), which is processed through roasting [173], and among others is Anglesite (PbSO_4_) [174]. Bioleaching using *A. ferrooxidans* and *A. thiooxidans* is a noteworthy alternative to conventional roasting and smelting processes for lead extraction [73]. The precipitation of Pb^2+^, released by galena as lead sulfate, concurs with elemental sulfur and sulfides undergoing oxidation by *A. ferrooxidans*, resulting in SO_4_^2−^ [175], as can seen from Equations (40)–(45):

PbS + H_2_SO_4_ + 0.5O_2_ → PbSO_4_ + H_2_O + S^0^ (40)

2S^0^ + 2H_2_O + 3O_2_ → 2SO_4_^2−^ + 4H^+^ (41)

PbS + 2Fe^3+^ → Pb^2+^ + 2Fe^2+^ + S^0^ (42)

Pb^2+^ + SO_4_^2−^ → PbSO_4_ (43)

PbS + H_2_SO_4_ → PbSO_4_ + H_2_S (44)

2H_2_S + O_2_ → 2S^0^ + 2H_2_O (45)

*A. ferrooxidans* and microbial consortia can facilitate the biorecovery of lead through various mechanisms such as the ones shown in Equations (40)–(45) and through biosorption, bioaccumulation, and biomineralization, effectively reducing the toxicity and mobility of lead in contaminated environments.

## 7. Metalloids: Arsenic

Arsenic (As) exhibits a pervasive presence, ranking 20th in abundance within the Earth’s crust, 14th in seawater, and emerging as the 12th most prevalent element within the human body [64]. Since its initial discovery, As has found diverse applications encompassing medicine, agriculture, metallurgy, and even as a potent poisonous substance for various organisms [176]. Arsenic is a known toxicant; nevertheless (1) it holds therapeutic potential as a frontline chemotherapeutic agent against specific hematopoietic cancers [177]; (2) some prokaryotes thrive and endure elevated As concentrations [178]. Among the mineral deposits, sulfide mineral realgar (alpha-As_4_S_4_) stands out due to its notably high arsenic content [179].

Among metalloids, arsenic is efficiently mobilized by *A. ferrooxidans*, which exhibits resilience to arsenic concentrations in the gram per liter range—an amount considered poisonous for numerous organisms. The catalytic conversion of Fe^2+^ to Fe^3+^ in an acidic solution instigates the generation of cationic As, as illustrated from Equations (46)–(50) [180]:

4Fe^2+^ + O_2_ + 4H^+^ → 4Fe^3+^ + 2H_2_O (46)

As_2_S_2_ + 6Fe^3+^ → 2As^3+^ + 2S^0^ + 6Fe^2+^ (47)

H_3_AsO_3_ + H_2_O → H_3_AsO_4_ + 2H^+^ + 2e^−^ (48)

H_3_AsO_4_ + Fe^3+^ → FeAsO_4_ + 3H^+^ (49)

In the presence of *A. ferrooxidans*:

As_2_S_2_ + 14H_2_O → 2H_3_AsO_3_ + 2HSO_4_^−^ + 20H^+^ +18e^−^ (50)

Bioleaching of arsenic and precipitation and oxidation of arsenite or reduction to arsenate are some valid processes involved in biorecovery activities and in treating contaminated environments such as AMDs, industrial wastewater, and polluted soils.

## 8. Actinides: Uranium

Uranium (U) is the only known actinide to be mobilized by *A. ferrooxidans*. The actinides, or actinide series, elements with atomic numbers ranging from 89 to 103, are situated beneath the lanthanide series. From uranium to lawrencium, each actinide brings its own set of intriguing features, making the study of this series crucial for understanding nuclear reactions, reactor design, and the behavior of radioactive materials [181].

Uranium finds applications in nuclear energy generation, the production of uranium oxide (U_3_O_8_), the formulation of anti-corrosive alloys, and coloring agents for glass and porcelain [74]. The conventional extraction of uranium relies on a process that utilizes acids as leaching agents [75]. Extracting uranium from low-grade ores through chemical leaching is often economically unfeasible [182]. In contrast, bioleaching is recognized for its economic viability and environmental compatibility [183]. *A. ferrooxidans* stands out in bioleaching for uranium extraction, leveraging its indirect oxidation capabilities as in Equation (51) [76,184]:

U^(IV)^O_2_ + 2Fe^3+^ → U^(VI)^O_2_^2+^ + 2Fe^2+^ (51)

Uranium exhibits limited solubility in an aqueous medium when it is in the +4 oxidation state. However, within an acidic environment, the ferric ion facilitates the oxidation of U^4+^ to U^6+^, a form that readily dissolves. In a complementary reaction to the oxidation of U^4+^, the ferric ion undergoes reduction to become the ferrous ion. Under the influence of *A. ferrooxidans* oxidation function, it is subsequently re-oxidized back to the ferric state. This enables it to once again engage in the oxidation of uranium [76]. Particularly, *A. ferrooxidans* sp. FJ2 is able to extract uranium by modulating the operon gene rus by 100% in 13 days [76]. The expression of some operons such as rus and petI, both involved in iron oxidation, are enhanced in the presence of radioactive minerals and after gamma-irradiation. Moreover, after exposure to gamma-rays, it was shown that this type of radiation does not affect the pH or the electrochemical parameters of the whole reaction, but increases the expression of all the genes encoded by the aforementioned operons without significant changes in uranium extraction rate [76].

## 9. Conclusions

**Figure 3:**
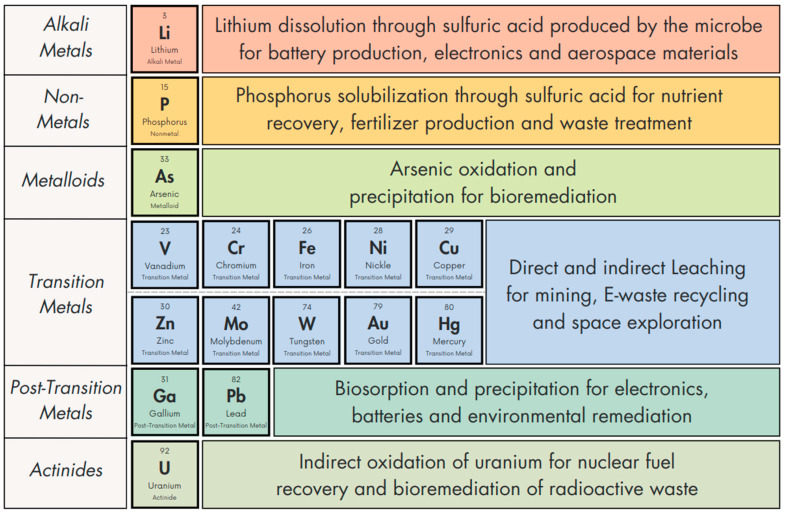
Periodic table of *A. ferrooxidans*-mobilized elements with main involved processes in elemental extraction techniques divided by chemical group. The periodic table of *A. ferrooxidans* is presented in bold.

*A. ferrooxidans* stands as a cornerstone in trace element bioleaching and in the biomining and biorecovery industry (Figure 3) [185]. Its specialized metabolic pathways, particularly in the oxidation of ferrous iron and sulfur compounds, combined with its ability to thrive and contribute to hyperacidic conditions, empower the efficient solubilization of diverse valuable trace elements from mineral deposits [12]. This microorganism has demonstrated remarkable proficiency in boosting metal (including alkali and transition metals, Figure 2) recovery rates, revolutionizing conventional mining methodologies. Its use significantly reduces the environmental footprint of mining operations, representing a critical step towards sustainable and eco-friendly mining practices [185]. This underscores the transformative potential of *A. ferrooxidans* in the journey toward sustainability [14], increasing our ability to recover critical metals from low-grade ore deposits, recycle E-wastes, and bioremediate contaminated sites.

Peering into the future, the application of *A. ferrooxidans* holds exciting prospects in space exploration. Its unique adaptability and capacity to facilitate metal extraction on Earth represents a groundbreaking future opportunity for resource utilization on other planetary bodies. By mitigating the challenges of resource transport from our home planet, the use of *A. ferrooxidans* may support future space missions, catalyzing the extraction of key elements for the development of extraterrestrial habitats and advancing technological frontiers [11].

One major concern with bioleaching and biomining is the production of acid mine drainage, a process that leads to significant environmental challenges [39]. However, bioleaching operations can be carefully controlled and optimized to minimize contamination risks and mitigate environmental impacts. Strategies such as monitoring pH, optimizing microbial consortia, and designing contained bioreactors or controlled leaching systems ensure that bioleaching processes can be sustainable. To counteract these negative impacts, it can be useful to employ biorecovery strategies, e.g., the usage of either naturally occurring or deliberately introduced microorganisms to consume and break down environmental pollutants, which are considered essential [186]. The effectiveness of biomining relies on the intricate interactions among the microbial communities involved. A deep comprehension of these interactions is strictly related to the effective management and optimization of the microbial consortia to enhance biomining efficiency [187], since the majority of current biomining activities use naturally occurring microbial populations [188]. It is worth noting that the dangers associated with the discharge of the bioremediating microorganisms into the surrounding environment to counteract acid mine drainage production are considered minimal, because these organisms are already present in the ecosystem [189,190]. As a consequence, while bioleaching and biomining present forward-thinking solutions for extracting metals, it is important to address their environmental repercussions and ensure sustainable resource recovery, aligning with the objectives of a circular economy [14].

The application of bioleaching to E-waste holds significant potential for enhancing social development and bolstering the economies of developing nations. The utilization of mesophilic and acidophilic/hyperacidophilic bacteria, particularly *A. ferrooxidans*, represents a substantial stride towards advancing base metal recovery both on Earth and potentially in extraterrestrial environments, e.g., planets (Mars), moons (Moon, Enceladus, Europa), and asteroids (Lutetia, Kleopatra). As we delve deeper into the intricacies of microbial-driven biomining processes, it is imperative to acknowledge the multifaceted contributions of *A. ferrooxidans*. From bolstering metal recovery rates on Earth to potentially revolutionizing resource extraction in space, this remarkable bacterium exemplifies the transformative potential of biotechnology in mining and beyond. With ongoing research and technological advancements, we are poised to further harness the capabilities of *A. ferrooxidans*, propelling us toward a future marked by sustainability, innovation, and abundant resources.

## Figures and Tables

**Table 1:** List of main elements, minerals, chemical compositions, and chemical reactions involved in bioleaching processes through *A. ferrooxidans*.

Element	Mineral	Chemical Formula	Reactions
Li	Lepidolite	KLi_2_Al(Si_4_O_10_)(F,OH)_2_	3Li_2_O*2K_2_O*5Al_2_O_3_*10SiO_2_*2SiF_4_ + 20H_2_SO_4_ → 3Li_2_SO_4_ + 2K_2_SO_4_ + 5Al_2_(SO_4_)_3_ + 11SiO_2_ + H_2_SiF_6_ + 18H_2_O + 2HF
P	P-Sludge	Ca_3_(PO)_4_	3H_2_SO_4_ + Ca_3_(PO_4_)_2_ → 2H_3_PO_4_ + 3CaSO_4_
V	V-Ti-Magnetite and Vanadates	(V,Ti),Fe^2+^Fe^3+^_2_O_4_ and V_2_O_3_	V_2_O_3_ + 2Fe^3+^ + 2H^+^ →2VO^2+^ + 2Fe^2+^ + H_2_O VO_2_ + Fe^3+^ → VO_2_^+^ + Fe^2+^ 10VO_2_ + 10Fe^3+^ + 8H_2_O → HV_10_O_25_^5−^ + 10Fe^2+^ + 15H^+^
Cr	Cr-Slug	Ca_3_Al_2_(H_4_O_4_, CrO_4_)_3_	Cr^6+^ + 3Fe^2+^ → Cr^3+^ + 3Fe^3+^
Ni	Ni-Sludge	Ni^0^	Fe_2_(SO_4_)_3_ + Ni^0^ → Ni^2+^ + SO_4_^2−^ + 2FeSO_4_
Cu	Chalcopyrite	CuFeS_2_	CuFeS_2_ + 2Fe_2_(SO_4_)_3_ → CuSO_4_ + 5FeSO_4_ + 2S^0^ CuFeS_2_ + 2H_2_O + 3O_2_ → Cu^2+^ + Fe^2+^ + 2H_2_SO_4_ CuFeS_2_ + H_2_SO_4_ → Cu^2+^ + FeSO_4_ + 2H^+^ + 2S^0^ CuFeS_2_ + 4H^+^ + O_2_ → Cu^2+^ + Fe^2+^ + 2H_2_O + 2S0 3FeSO_4_ + H_2_SO_4_ + 0.5O_2_ → Fe_2_(SO_4_)_3_ + FeSO_4_ + H_2_O
As	Realgar	As_2_S_2_ or alpha As_4_S_4_	4Fe^2+^ + O_2_ + 4H^+^ → 4Fe^3+^ + 2H_2_O As_2_S_2_ + 6Fe^3+^ → 2As^3+^ + 2S^0^ + 6Fe^2+^ H_3_AsO_3_ + H_2_O → H_3_AsO_4_ + 2H^+^ + 2e^−^ H_3_AsO_4_ + Fe^3+^ → FeAsO_4_ + 3H^+^ As_2_S_2_ + 14H_2_O → 2H_3_AsO_3_ + 2HSO_4_^−^ + 20H^+^ +18e^−^
Mo	Mo Sulfide	MoS_2_	4MoS_2_ + 4Fe^3+^ + 16H_2_O → 4MoO_4_^2−^ + 4Fe^2+^ + 32H^+^ + 8S^0^
W	Tungstate Salts	WO_4_^2−^	H_2_SO_4_ + FeWO_4_ → FeSO_4_ + H_2_WO_4_
Au	Au Ores	Au^0^	4Au^0^ + 8NaCN + 2H_2_O + O_2_ → 4NaAu(CN)_2_ + 4NaOH
Hg	Cinnabar	HgS	HgS + 2Fe^3+^ → Hg^2+^ + 2Fe^2+^ + S^0^ 2HgS + 4Fe^3+^ +1.5O_2_ +2H_2_O → 2Hg^2+^ + 4Fe^2+^ + 2H_2_SO_4_
U	U Oxide	UO_2_	U^(IV)^O_2_ + 2Fe^3+^ → U^(VI)^O_2_^2+^ + 2Fe^2+^

**Table 2:** Selected bioleached elements by *A. ferrooxidans*. A = *Acidithiobacillus ferrooxidans*, B = other organisms, and C = consortia. 1. According to the literature, the estimated copper extraction through biohydrometallurgy is about 30% worldwide. For the other metals, there are no clear data in the literature. * All the biomining experiments made in space refer to microgravity conditions on the ISS or simulated environments. d—estimates according to British Geological Survey.

Element	Main Alloys	Application Fields	Metal Printing	Electronics	Extraction Yield (ktons/year) ^d^	Biomining on Earth	Biomining in Space *	Biorecovery	References
Ti	Alpha/BetaTi alloys	Aerospace, Construction, Automobile	Yes	Major (hardware)	6300	Yes (A)	No	Yes (A,B,C)	[52]
V	Ferrovanadium	Structural, Fusion Reactor	No	Minor (semiconductors)	81	Yes (A)	Yes (B)	Yes (A,B,C)	[9,10,53,54,55,56]
Cr	FeCr, Stainless steel	Automobile, High Temperature	Yes	Minor (anticorrosive coating)	38,600	Yes (A)	No	Yes (A,B,C)	[43,57,58,59]
Fe	Steel, Inox	Construction, Automobile, Aerospace,	Yes	Major (solder)	3,040,000	Yes (A,B,C)	Yes (B)	Yes (A,B,C)	[9,10,22]
Ni	NiCrFe	Medical, Aerospace, Energy, Isolators, Automotive, Cables	Yes	Minor (plating agent)	2700	Yes (A)	Yes (B)	Yes (A,B,C)	[9,10,60]
Cu	Brass, Bronze	Construction, Marine, Automotive, Medical, Aerospace	Yes	Major (cables)	20,700	Yes (A,B,C) 1	Yes (B)	Yes (A,B,C)	[9,10,61]
Ga	GaAlZn alloys	Semiconductors, Diodes, Circuits	No	Major (diodes)	0.380	Yes (A)	No	No	[62,63]
As	As Bronze	Automobile, Ammunition	No	Minor (batteries)	NA	Yes (A,B,C)	No	Yes (A,B,C)	[44,64]
W	W,Ni,Cu and W,Ni,Fe alloys	Aerospace, Medical, Automobile	Yes	Minor (light bulbs)	91.5	Yes (A)	No	Yes (A,B,C)	[65,66,67]
Au	AuPt alloys	Increasing Corrosion Resistance	No	Major (connector)	3.3	Yes (A)	No	No	[68,69,70,71]
Pb	PbCu, alloys	Automobile, Ammunition, Batteries	No	Major (solder)	4700	Yes (A,C)	No	Yes (A,B,C)	[72,73]
U	Mulberry	Increasing Corrosion Resistance	No	None	53	Yes (A)	No	Yes (A,B,C)	[74,75,76]

## Data Availability

No new data were created.
